# Proteomic Identification of Calumenin as a G551D - CFTR Associated Protein

**DOI:** 10.1371/journal.pone.0040173

**Published:** 2012-06-29

**Authors:** Ling Teng, Mathieu Kerbiriou, Mehdi Taiya, Sophie Le Hir, Olivier Mignen, Nathalie Benz, Pascal Trouvé, Claude Férec

**Affiliations:** 1 Inserm, UMR1078, Brest, France; 2 Université de Bretagne Occidentale, Faculté de Médecine et des sciences de la santé, Brest, France; 3 Université de Bretagne Occidentale, Service commun de spectrométrie de masse, Brest, France; 4 C.H.R.U. Morvan, Laboratoire de Génétique Moléculaire, Brest, France; 5 Etablissement Français du Sang, Brest, France; 6 Association de Biogénétique Gaëtan Salaün, Brest, France; St. Georges University of London, United Kingdom

## Abstract

Cystic fibrosis (CF) is the most common lethal autosomal recessive disease in the Caucasian population. It is due to mutations in the cystic fibrosis transmembrane conductance regulator (CFTR) gene. To date, over 1910 mutations have been identified in the CFTR gene. Among these mutations, the CF-causing missense mutation G551D-CFTR (approx. 5% of cases) encodes for a CFTR chloride channel with normal expression on the cell surface. Nevertheless, it is associated with severe disease due to its altered channel activation. The aim of the present study was to identify specific interacting proteins of G551D-CFTR. Co-immunoprecipitated proteins with G551D-CFTR were resolved by 2D-gel electrophoresis (2-DE). Mass Spectrometry revealed that calumenin was present in the protein complex linked to G551D-CFTR. Despite its basal expression was not modified in G551D-CFTR expressing cells when compared to Wt-CFTR expressing cells, it was more abundant in the G551D-CFTR complex detected by immunoprecipitation. The calumenin-CFTR interaction was also shown by Surface Plasmon Resonance and further confirmed by computational analysis of the predicted calumenin’s partners. Because in our cellular model calumenin was found in the endoplasmic reticulum (ER) by immunofluorescence experiments, we suggest that calumenin is likely involved in the mutated CFTR’s maturation. In conclusion, we showed for the first time that calumenin binds to CFTR and that it is increased in the G551D-CFTR complex. We suggest that it may be involved in the physiopathology of G551D-CFTR and that G551D-CFTR may follow a specific maturation and trafficking pathway. We also hypothesize that UPR may be triggered independently of the retention of G551D-CFTR in the ER because Grp78/Bip expression is increased in the cells. Finally, we propose here that Calumenin is a new CFTR chaperone.

## Introduction

Cystic fibrosis (CF) is the most common lethal autosomal recessive disease in the Caucasian population. It is due to mutations in the cystic fibrosis transmembrane conductance regulator (CFTR) gene [1]–[3]. CFTR is an ATP-binding cassette transporter functions as a chloride (Cl^-^) channel [4]–[6] and comprises two hydrophobic core regions, two nucleotide-binding domains (NBDs) with ATP-binding activity [7] and a regulatory domain (R domain). CFTR channel opening requires phosphorylation by cAMP-dependent protein kinases (PKA) [8] and hydrolyzable MgATP [9], [10]. Both genetic and interacting proteins seem to be involved in the CFTR regulation. Indeed, CFTR regulation is complex and involves dimerization of the protein [11], [12] and interdomain interactions [13]. Syntaxin 1A, EBP50, E3KARP, the µ subunit of the endocytic clathrin adaptor complex, cysteine string proteins and annexin A5 are CFTR-binding proteins [14]–[19], but the extent to which CFTR channels are regulated by protein-protein interactions remains largely unknown.

To date, over 1910 mutations (www.genet.sickkids.on.ca/cftr/) have been identified in the CFTR gene and a classification of mutations by which different mechanisms induce CF has been proposed [20]. Among these mutations, the CF-causing missense mutation G551D-CFTR (Gly to Asp at position 551) exhibits normal expression at the cell surface but it is associated with severe disease [21], [22]. Indeed, it lacks channel activation mediated by ATP [23], [24]. G551D-CFTR is not a common mutation in the CF patients (approx. 5% of cases) but the clinical phenotype is considered very severe. Therefore, efforts have been taken to overcome the G551D-CFTR defect. Biochemical studies on purified and reconstituted G551D-CFTR showed the potentiation of the ATPase activity by VRT-532 [25]. Nevertheless, VRT-532 did not affect the ATPase activity of the Wt (wild-type) CFTR. This supported the idea that this compound corrects the specific molecular defect of this mutant by a direct or indirect binding, stabilizing an intramolecular interaction. This potentiator seems to have a mutant specificity which may be due to CFTR interacting proteins [26].

As it is suggested that G551D-CFTR has different binding partners when compared to Wt-CFTR, the aim of the present study was to identify specific interacting proteins of G551D-CFTR. The proteins linked to G551D-CFTR were resolved by 2D-gel electrophoresis (2-DE). Among the detected spots, one spot exhibited a high intensity and was subjected to Mass Spectrometry (MS). MS revealed that the corresponding protein was calumenin. We found that its basal expression was not modified in G551D-CFTR expressing cells when compared to Wt-CFTR expressing cells. Using co-immunoprecipitation, we found that calumenin was bound to the G551D-CFTR protein. The co-immunoprecipitation experiment also indicated that calumenin was also bound to Wt-CFTR. Nevertheless, the amount of bound calumenin was higher in the G551D-CFTR complex than in the Wt-CFTR one. The calumenin - CFTR interaction was further confirmed by computational interaction prediction and by Surface Plasmon Resonance (SPR). In order to know whether calumenin was localized in the Endoplasmic Reticulum (ER) in our cell model and whether the interaction takes place in the ER in which calumenin is known to be present, immunofluorescence was performed. We found that the calumenin - CFTR interaction is indeed only present in the ER of the cells. Because calumenin expression is modulated in cells expressing the most frequent CFTR mutant (F508del) together with Grp78/Bip which is a hallmark of the Unfolded Protein Response (UPR) and because we found an increased level of calumenin linked to G551D-CFTR, we assessed the Grp78 expression in the cells. We found that Grp78 expression was increased in G551D-CFTR expressing cells, suggesting that calumenin is likely involved in the maturation of CFTR within the ER, that G551D-CFTR may follow a specific maturation and trafficking pathway and that UPR may be triggered in CF independently of the retention of G551D-CFTR in the ER. Finally, we propose that Calumenin is a new CFTR’s chaperone.

## Results and Discussion

**Figure 1:**
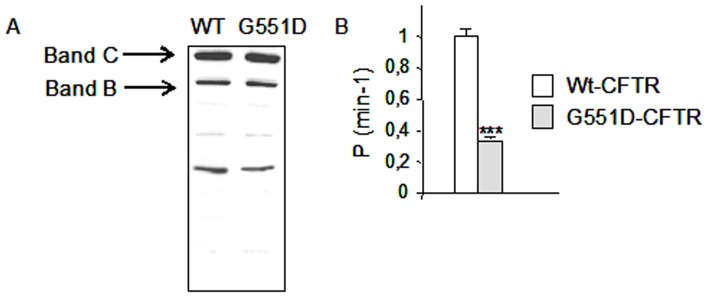
Validation of the cell model. **A,** Wt-CFTR and G551D-CFTR expression in Hela cells. Example of western blot showing the mature fully glycosylated CFTR and core-glycosylated CFTR labeled as Band C (170 kDa) and Band B, respectively. **B,** Study of the cyclic AMP-induced I^−^ efflux from Wt-CFTR (n = 3) and G551D-CFTR (n = 3) using SPQ experiments. The halide permeability (p) is the rate of SPQ dequenching. The graphs show a deeply decreased activity in G551D-CFTR expressing cells. Data are the means ± S.E.M., ***: p<0.001.

Wt-CFTR and G551D-CFTR were expressed in Hela cells which do not endogenously express CFTR and their function was measured by [27], [28]. The expression of the CFTR protein was assessed by western blotting. As shown in Fig. 1A the mature fully glycosylated CFTR and core-glycosylated CFTR were expressed in both cell types. Therefore, we found that in our cell model the mutant protein can fold and traffic normally to the plasma membrane. This was in accordance with previous results showing that G551D-CFTR exhibits no trafficking defects [23], [29]. We investigated the cAMP-dependent halide flux through Wt-CFTR and G551D-CFTR (Fig. 1B) in the stably transfected cells. We found that it was deeply decreased in G551D-CFTR cells, as previously described [9], [29]. Therefore, we showed that viral tranfection of the cells led to the expression of Wt-CFTR and G551D-CFTR and that their expression and function were in accordance with previous results, indicating that the cell model could be used for further experiments.

**Figure 2:**
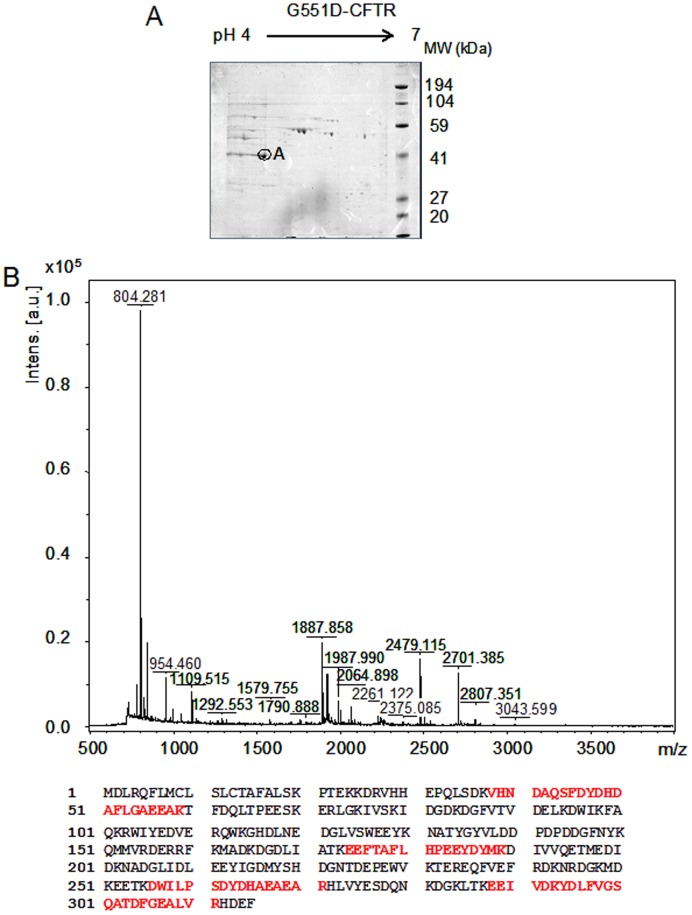
Calumenin belongs to the G551D-CFTR’s complex. **A,** Example of a reference 2-DE (pH 4–7) of 6 gels performed with immunoprecipitated G551D-CFTR. The image analysis detected 165 protein spots. The analyzed spot is noted as A. **B,** MS spectra obtained for the A spot and protein coverage (24%) shown in bold red on the calumenin amino acid sequence (gi/2809324).

A reference 2-DE (pH 4–7) of 6 gels performed with proteins complex linked to G551D-CFTR obtained by immunoprecipitation is presented in Fig. 2A. The image data analysis detected 165 protein spots in the complex. The spot showing the higher expression was further analyzed (A, on Fig. 2A). It was excised from the gel, trypsined and analyzed by MS. The results indicate that the spot corresponded to calumenin (table 1). The MS/MS results leading to calumenin identification are summarized in table 2. The MS spectrum and the coverage of the calumenin sequence are presented in Fig. 2B.

**Table 1:** Identification of spots A by Mass Spectrometry.

Spot	RefSeqAC	Protein Name	Gene	Sequence Coverage	Queries Matched	Molecular weight/Isoelectric point	Observed in (cell type)
A	NM001219	Calumenin	CALU (Homo sapiens)	67	66	37084/4.47	G551D-CFTR

**Table 2:** MS/MS results for Calumenin identification.

Tree hierarchy	Meas. M/z	Calc. MH+	Meas. Mr	Calc. Mr	Int.	Dev.(ppm)	Score	Mascot Score	Range
MSMS 25	1887,858	1887,851	1886,851	1886,843	18320,6	3,746	25	81	256–271
MSMS 38	2064,898	2064,89	2063,891	2063,882	3936,262	4,134	7	7	174–189
MSMS 50	2479,115	2479,08	2478,108	2478,072	11680,34	14,325	89	109	38–59
MSMS 55	2701,385	2701,336	2700,378	2700,328	8222,202	18,254	2059	152	288–311

*Search Parameter: Charge = 1+, Trypsin, Mascot 2.3.01.241, NCBInr NCBInr_20120204.fasta; Modifications: Global: Carboxymethyl (C), Optional: Oxidation (M). Individual ions scores >51 indicate identity or extensive homology (p<0.05). Protein scores are derived from ions scores as a non-probabilistic basis for ranking protein hits.*

**Figure 3:**
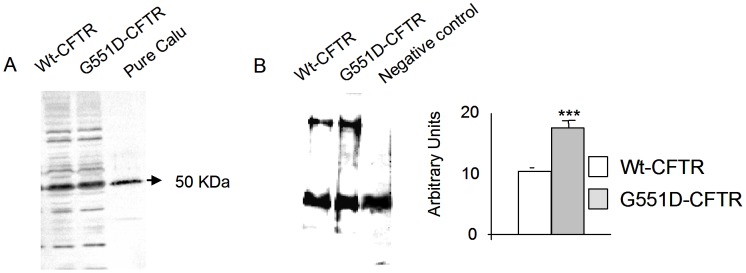
More calumenin is bound onto G551D-CFTR than onto Wt-CFTR. **A,** Example of detection of calumenin in Wt-CFTR and G551D expressing cells, assessed by western blotting using whole cell lysates. No difference was observed between the two cell lines. Pure Calumenin was loaded as a molecular weight control because of the presence of non-specific bands on the membrane. **B,** Example of coimmunoprecipitation showing the calumenin-CFTR interaction in total proteins (left). Histogram represents the quantitation indicating that more calumenin is bound onto G551D-CFTR than onto Wt-CFTR (right).

Calumenin is a multiple EF-hand Ca^2+^-binding protein located in the ER. It belongs to the CREC family of Ca^2+^-binding proteins. It is also found in the secretory pathway and can be secreted to the extracellular space. It interacts with different ligands and it inhibits several proteins in the ER membrane such as the ryanodine receptor (RyR2) and the Ca^2+^-transporting ATPase (SERCA2). Some other functions concern participation in the secretory process, ER lumenal chaperone activity and signal transduction. It is also involved in a large variety of disease processes (for review [30]) and it was shown to be involved in the Ca^2+^ homeostasis of the ER, due to a direct interaction with SERCA2 and RyR2. Indeed, an inhibition of SERCA2-mediated Ca^2+^ uptake into SR in cardiomyocyte due to an interaction with calumenin was observed [31]. To assess the expression of calumenin in G551D-CFTR expressing cells, western blots (n = 5) were performed to compare its basal level with that of wt-CFTR expressing cells. As shown in Fig. 3A, no difference was observed between Wt- or G551D-CFTR expressing cells suggesting that it is not involved in a difference of the Ca^2+^ homeostasis between Wt- and G551D-CFTR cells. Whereas, calumenin was detected in the CFTR’s ER-Associated Folding Proteome and seems to be modulated in Fdel508-CFTR expressing cells, its direct interaction with CFTR has never clearly been established [32], [33]. Therefore, we assessed its interaction with CFTR by coimmunoprecipitation. As shown in Fig. 3B, we found that calumenin was bound to CFTR and that it was more abundant in the G551D-CFTR complex than in the Wt-CFTR complex. The reverse coimmunoprecipitation indicated that the amount of bound G551D-CFTR onto calumenin was higher than the amount of bound Wt-CFTR (not shown). Because we found that calumenin binds to the mature G551D-CFTR form (170 kDa) and because human calumenin is found in the ER as well as in the Golgi complex [34], our results suggest that it is likely involved in the CFTR maturation.

**Figure 4:**
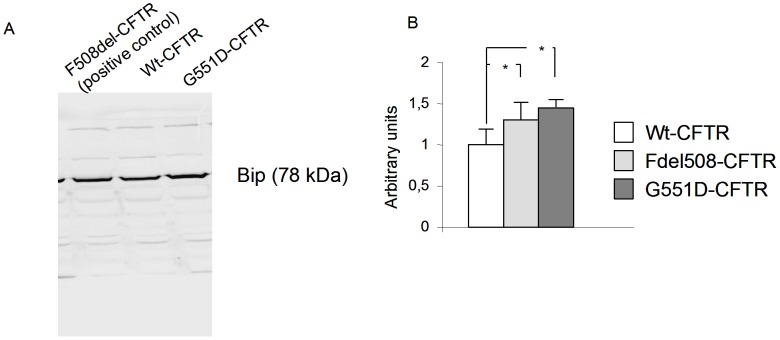
UPR is likely triggered in G551D-CFTR expressing cells. **A,** Example of detection of Grp78 in Wt-CFTR, Fdel508-CFTR and G551D-CFTR expressing cells, assessed by western blotting using whole cell lysates. A Fdel508-CFTR expressing cell lysate was used as a positive control. **B,** Histogram represents the quantitation indicating that the amount of Grp78 is increased in Fdel508-CFTR and G551D-CFTR expressing cells whereas no difference is observed between Fdel508-CFTR and G551D-CFTR expressing cells (right).

Grp78/Bip is a major sensor for the Unfolded Protein Response (UPR) triggering in the ER. It is modulated as well as calumenin in F508del-CFTR expressing cells and we previously showed that it is involved in the observed UPR in CF cells [33], [35]. Therefore, we assessed Grp78 expression in G551D-CFTR expressing cells, using Fdel508-CFTR cells as a positive control. As shown in Fig. 4A, we observed an increased Grp78 expression in G551D-CFTR cells when compared to Wt-CFTR expressing cells. The quantitation indicated that its expression was not significantly different to that observed in Fdel508-CFTR cells, suggesting the UPR triggering (Fig. 4B). Because it was previously shown that G551D-CFTR is not retained in the ER [36] this result suggests that UPR, or at least an ER stress, may be triggered in G551D-CFTR expressing cells due to another mechanism involving calumenin.

**Figure 5:**
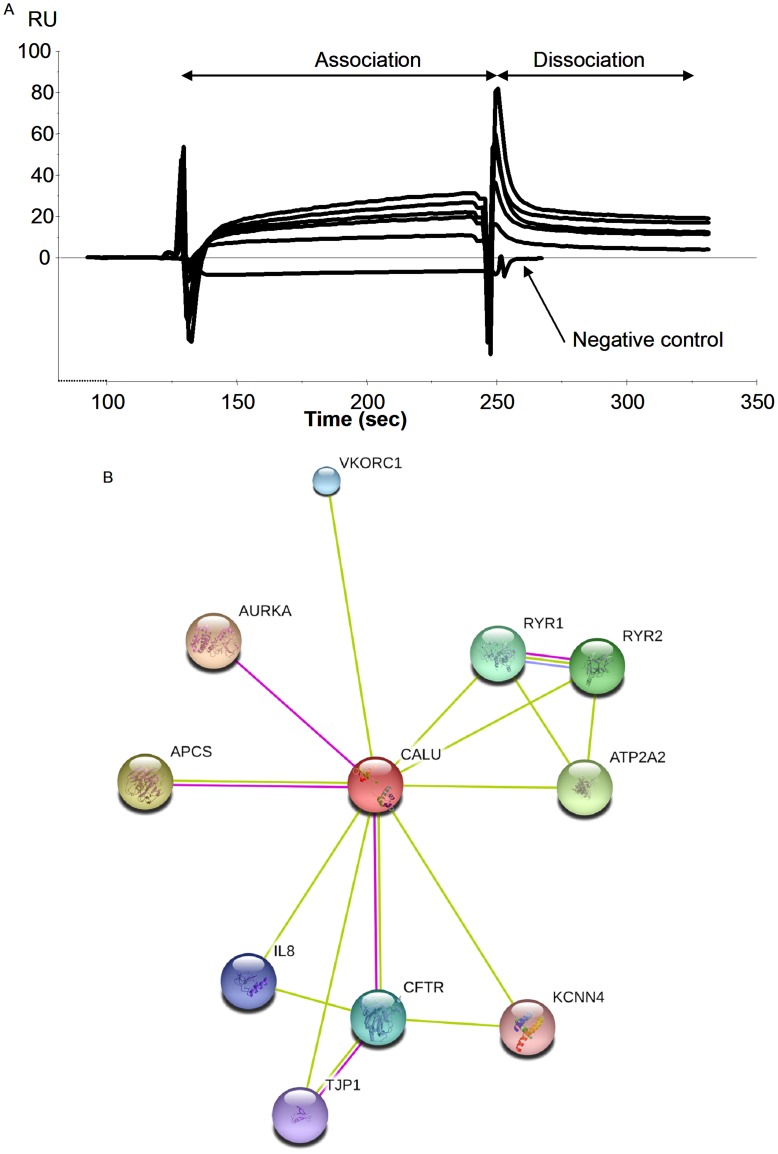
Evidence of the direct calumenin-CFTR interaction. **A,** Example of sensorgrams obtained when pure Calumenin was linked on the sensorchip and pure CFTR was injected (0.15 to 1.5 nM), showing an interaction followed by a dissociation phase which was not zero when the injection was stopped. The obtained value for the dissociation constant was 3.8e−12 M showing a strong interaction. SPR was performed to rule out a possible non specific binding in co-immunoprecipitation experiment. **B,** Network of the predicted interactions for Calumenin obtained by STRING. Green connecting lines indicates that the interaction in mentioned in PubMed abstracts with no or insignificant experimental data. Pink connecting lines indicates that the interaction has been demonstrated with available biochemical data. A pink line was observed for the Calumenin - CFTR interaction [32]. Partners are depicted in Table 3.

**Figure 6:**
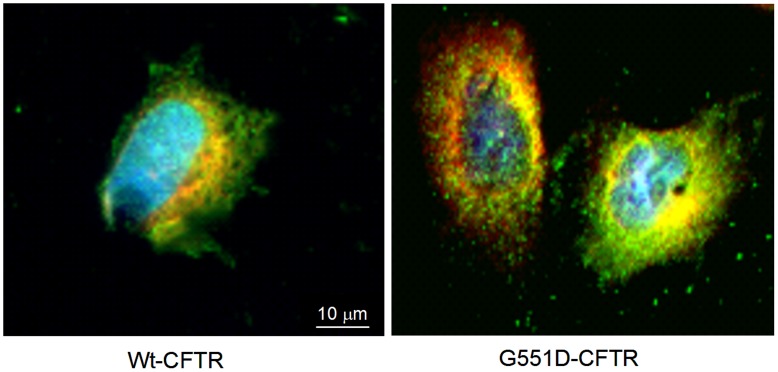
The calumenin – CFTR interaction takes place in the ER. Immunofluorescent localization of Calumenin within Wt- (left) and G551D-CFTR (right) expressing cells. The nuclei were labelled in blue by DAPI. The ER was visualized using PDI labelling (red) and Calumenin was seen in green. Overlapping of PDI and Calumenin is yellow showing the described interaction between Calu and CFTR takes place within the ER.

Because the calumenin - CFTR interaction in the ER of cells had never been shown before and because some extra bands were observed when calumenin was detected in the cells using western blottings, we had to further provide evidence showing this interaction. For this purpose, Surface Plasmon Resonance was used [37]. The sensorgrams obtained when pure calumenin was linked on a sensorchip and pure CFTR was injected (0.15 to 1.5 nM), showing an interaction followed by a dissociation phase which was not zero when the injection was stopped indicated that the interaction occured (Fig. 5A). The obtained value for the dissociation constant was 3.8×10^−12^ M showing a strong interaction. The opposite experiment in which purified CFTR was linked on the sensorchip was also performed (not shown). The SPR experiments confirming the direct interaction permitted to rule out the possibility of an unspecific binding during co-immunoprecipitation experiments. The calumenin - CFTR interaction was also evaluated with the online Search Tool for the Retrieval of Interacting Genes/Protein (STRING 9.0, http://string-db.org) to predict both direct and indirect interactions [38]. As shown in Fig. 5B CFTR was found to be a calumenin’s network. Among the predicted calumenin’s partners which are listed in Table 3, some are depicted and characterized by biochemical experiments such as RyR and ATP2A2 [30], [31]. In order to show that in our cell model calumenin is located within the ER, as described in other cell types, immunofluorescence was performed. The nuclei of the cells as well as the ER and calumenin were labelled in wt- and G551D-CFTR expressing cells (Fig. 6). The results indicated that in our cell model calumenin is indeed localized in the ER and that therefore the described interaction between Calumenin and wt- or G551D-CFTR takes place within the ER.

**Table 3:** Predicted Functional Partners for Calumenin.

Name	Ensembl project number(http://www.ensembl.org)	Role	Interactionscore (STRING)
AURKA	*ENSP00000216911*, Homo sapiens	May play a role in cell cycle regulation during anaphase and/or telophase,in relation to the function of the centrosome/spindle pole region duringchromosome segregation. May be involved in microtubule formationand/or stabilization.	0.974
APCS	*ENSP00000255040*, Homo sapiens	Amyloid P component, serum. Can interact with DNA and histones and may scavenge nuclear material released from damaged circulating cells. May also function as a calcium-dependent lectin	0.929
ATP2A2	*ENSP00000324892*, Homo sapiens	ATPase, Ca^2+^ transporting, cardiac muscle, slow twitch 2. This magnesium-dependent enzyme catalyzes the hydrolysis of ATP coupled with thetranslocation of calcium from the cytosol to the sarcoplasmic reticulumlumen. Isoform SERCA2A is involved in the regulation of thecontraction/relaxation cycle	0.860
RYR2	*ENSP00000355533*, Homo sapiens	ryanodine receptor 2 (cardiac); Communication between transverse-tubulesand sarcoplasmic reticulum. Contraction of cardiac muscle is triggered byrelease of calcium ions from SR following depolarization of T- tubules(By similarity)	0.853
RYR1	*ENSP00000352608*, Homo sapiens	ryanodine receptor 1 (skeletal); Communication between transverse-tubulesand sarcoplasmic reticulum. Contraction of skeletal muscle is triggered byrelease of calcium ions from SR following depolarization of T-tubules	0.815
**CFTR**	*ENSP00000003084*, Homo sapiens	cystic fibrosis transmembrane conductance regulator (ATP-binding cassettesub-family C, member 7); Involved in the transport of chloride ions. Mayregulate bicarbonate secretion and salvage in epithelial cells by regulatingthe SLC4A7 transporter	0.801
VKORC1	*ENSP00000378426*, Homo sapiens	vitamin K epoxide reductase complex, subunit 1; Involved in vitamin K metabolism. Catalytic subunit of the vitamin K epoxide reductase (VKOR)complex which reduces inactive vitamin K 2,3-epoxide to active vitamin K	0.778
IL8	*ENSP00000306512*, Homo sapiens	interleukin 8; IL-8 is a chemotactic factor that attracts neutrophils, basophils,and T-cells, but not monocytes. It is also involved in neutrophil activation. It isreleased from several cell types in response to an inflammatory stimulus.IL-8(6–77) has a 5–10-fold higher activity on neutrophil activation,IL-8(5–77) has increased activity on neutrophil activation and IL-8(7–77)has a higher affinity to receptors CXCR1 and CXCR2 as comparedto IL-8(1–77), respectively	0.764
TJP1	*ENSP00000281537*, Homo sapiens	tight junction protein 1 (zona occludens 1); The N-terminal may be involvedin transducing a signal required for tight junction assembly, while theC-terminal may have specific properties of tight junctions. The alphadomain might be involved in stabilizing junctions	0.750
KCNN4	*ENSP00000262888*, Homo sapiens	potassium intermediate/small conductance calcium-activated channel,subfamily N, member 4; Forms a voltage-independent potassium channelthat is activated by intracellular calcium. Activation is followed bymembrane hyperpolarization which promotes calcium influx. The channel isblocked by clotrimazole and charybdotoxin but is insensitive to apamin	0.748

### Conclusion

Our results suggest that the G551D-CFTR’s maturation/trafficking is different from the one of Wt-CFTR because it likely involves more calumenin. Furthermore, we suggest that UPR is likely triggered in G551D-CFTR expressing cells. Nevertheless, the UPR pathway may be different than the one observed in Fdel508-CFTR cells because the G551D-CFTR protein is not retained in the ER. In conclusion, we show here a direct calumenin - wt-CFTR and calumenin - G551D-CFTR interaction. Because we found the interaction within the ER and because the amount of bound calumenin is likely higher with the mutated CFTR, we propose that the G551D-CFTR protein may be directed to a specific trafficking pathway. The exact role of calumenin upon UPR in G551D-CFTR expressing cells is now under investigation in our laboratory as well as its possible role as a chaperone for the most frequent mutation found in CF (F508del) which is retained in the ER. Furthermore, according to the Bip/Grp78 expression, we propose that UPR may be triggered in G551D-CFTR expressing cells, independently of any retention of the mutated CFTR in the ER.

## Materials and Methods

### Plasmid Construction

The pLenti6/V5-CFTR mammalian expression lentiviral construct, driven by the cytomegalovirus promoter was constructed using ViraPower™ Lentiviral Directional TOPO® Expression Kit (Invitrogen). To clone the cDNA encoding the human wild type CFTR (Wt-CFTR, Transgene SA, pTG5985, France, access Genbank no. M28668) into the pLenti vector, a forward primer sequence with 4 nucleotides CACC- at the 5′ end, based on the TOPO-cloning technology, was designed according to manufacturer’s instruction. The cDNAs encoding G551D-CFTR and F508del-CFTR were generated by site-directed mutagenesis (QuickChange II XL Site-Directed Mutagenesis Kit, Stratagene).

### Viral Particle Production and Cell Transfection

6x10^6^ 293FT cells (provided with the ViraPower™ Lentiviral Directional TOPO® Expression Kit, InVitrogen) were plated 24 hours prior to the addition of a complex comprising Wt-CFTR, G551D-CFTR or F508del-CFTR plasmid and Lipofectamine™ 2000 (*Invitrogen*). The HIV type 1 (HIV-1) *gag-pol* construct pLP1, *rev* construct pLP2 and the Vesicular Stomatitis Virus G glycoprotein (VSV-G) construct pLP/VSVG were delivered together with the pLenti expression vector into the cells at a ratio of 3∶3:3∶3 µg, respectively. Cells were washed 24 hours post-transfection and incubated with fresh media. Supernatants containing viral particles were harvested 72 hours post-transfection and titrated on HeLa cells. The remaining virus was stored at −80°C before use. The transfection of HeLa cells was performed in the presence of Polybrene (6 µg/ml, Chemicon). To transfect 2×10^5^ cells, 1 mL supernatants containing lentivirus was added (3×10^3^ TU/ml). Cells were incubated with fresh media 24 hours after transfection and were subsequently treated by Blasticitin for antibiotic selection 48 hours after transfection.

### Cell Culture

293FT cells and HeLa cells (from ATCC, N°CRM-CCL-2, USA) were cultured in Dulbecco’s Modified Eagle’s medium supplemented with 10% fetal bovine serum. HeLa cells stably expressing either Wt-CFTR, G551D-CFTR and F508del-CFTR were selected using Blasticitin (2 µg/ml) for at least 10 days.

### Protein Preparation and Immunoblot Analysis

Wt-CFTR, G551D-CFTR and F508del-CFTR expressing cells were collected and homogenized using a glass homogenizer in 2 mL of RIPA buffer. Lyzates were ultra-centrifuged (32500 rpm, 30 min., 4°C). The pellets were resuspended in 500 µL of RIPA. The Wt-CFTR, G551D-CFTR and F508del-CFTR expression were assessed by western blottings. Proteins (50 µg) were loaded on a Bis/Tris precasted polyacrylamide gel (10%, Invitrogen) and transferred onto a PVDF membrane. CFTR proteins were probed using a monoclonal anti-CFTR antibody (24-1, R&D System) and a horseradish peroxidase (HRP) conjugated secondary antibody (Amersham). Other used antibodies were polyclonal anti-calumenin antibody (D-19, Santa Cruz Biotechnology) and polyclonal anti-Grp78 antibody (H-129, Santa Cruz Biotechnology). Horseradish peroxidase (HRP) conjugated secondary antibodies were from Amersham. Blots were developed by enhanced chemiluminescence kit (ECL Plus, Amersham) and analyzed using Chemi-Smart 5100 ECL imaging system (Vilber Lourmat). Intensity of each band was quantified using densitometric analysis of the signals (GelDoc 2000, Biorad). Each value was normalized by the total amount of the loaded proteins per lane which was estimated by staining of the membranes by coomassie blue. Normalization was also performed by the actin signal obtained on the same membranes. The results did not vary with the normalization type.

### Methoxy-N-(3-sulfopropyl)quinolinium (SPQ) Fluorescence Assay

Stably transfected cells expressing either Wt-CFTR or G551D-CFTR were used in SPQ experiments as previously described, using forskolin as CFTR activator because it induces cAMP synthesis which regulates the CFTR chloride channel function [19], [27]. Cells were loaded with intracellular SPQ dye by incubation in Ca^2+^ -free hypotonic (50% dilution) medium containing 10 mM SPQ, 15 min, at 37°C. Coverslips were mounted on the stage of an inverted microscope equipped for fluorescence and illuminated at 360 nm. The emitted light was collected at 456+/−33 nm by a high-resolution image intensifier coupled to a video camera connected to a digital image processing board controlled by FLUO software (Imstar, France). Cells were maintained at 37°C and continuously superfused with an extracellular solution containing in mM:145 NaCl, 4 KCl, 1 MgCl2, 1 CaCl2, 5 HEPES and 5 glucose, pH 7.4. A microperfusion system allowed local application and rapid change of the different experimental mediums. Iodide (I^−^) and nitrate (NO3^−^) containing media were identical to the extracellular solution except that I^−^ and NO3^−^ replaced Cl^−^ as the dominant extracellular anions. All extracellular media also contained 10 µM bumetanide to inhibit the Cl^−/^Na+/K+ co-transporter. Single-cell fluorescence intensity was measured from digital image processing and displayed against time. Fluorescence intensity was standardized according to the equation F = (F−Fo)/Fo×100, where F is the relative fluorescence and Fo, the fluorescence intensity measured in presence of I^-^. The halides membrane permeability (p) was determined as the rate of SPQ dequenching upon perfusion with nitrates. At least three successive data points were collected immediately after the NO3^−^ containing medium application, and then fitted using a linear regression analysis. The slope of the straight line reflecting the membrane permeability to halides (noted p in min-1) was used as an index of CFTR activity [19], [27].

### Immunoprecipitation

Stably transfected HeLa cells expressing G551D-CFTR were washed in cold PBS, scraped into PBS, pelleted and resuspended in the RIPA buffer (25 mM Tris-HCl, 150 mM NaCl, 1% Triton X-100, 1% Na deoxycholate, 0.1% SDS, 10 mM iodoacetamide, 100 µM PMSF, pH 7.5) in the presence of fresh protease inhibitor cocktail (P8340, Sigma-Aldrich) at a final concentration of 4.5 µl/ml. Anti-CFTR monoclonal antibody M3A7 (Millipore) or anti-calumenin antibody was incubated with magnetic beads (Dyna beats Protein G, Invitrogen) at a final concentration of 0.2 mg per ml of wet beads at 4°C for 1 hour with rocking. 500 µg of whole cell lysate were applied to the beads-antibody complex. Beads were then incubated at 4°C overnight and washed 3 times in cold PBS. Immunoprecipitated proteins were subsequently eluted with 30 µl of SDS-PAGE loading buffer at 95°C for 10 min. Control experiments were performed without any antibody linked to the beads.

### Immunofluorescence

Cells were fixed in 4% PFA for 10 min. After fixation and three 5-min washes with PBS-T (PBS 1X plus Tween 0.1%), cells were permeabilized with 0.5% Triton X-100 in PBS for 4 min. After 5-min washes with PBS-T, nonspecific binding was prevented by blocking with 2% BSA in PBS 1X f (30 min, room temperature (RT)). Primary antibody against Calu (H-40, Santa Cruz; 1∶100 in BSA 2%) was applied for 2 h at RT. After three 5-min washes with PBS-T, cells were incubated for 1 h 30 at RT with Alexa fluor 488 goat anti-rabbit IgG (Molecular probes) secondary antibody diluted 1∶1000 in BSA 2%. Then, after three 5-min washes with PBS-T, a second labelling against PDI (Protein Disulfide Isomerase, Invitrogen) was performed. Primary antibody against PDI (1∶1000 in 2% BSA) was applied (12 h, 4°C). After three 5-min washes with PBS-T, cells were incubated for 1 h 30 at RT with Goat anti mouse texas red (Molecular probes) secondary antibody diluted (1∶1000 in BSA 2%). Nuclei were labelled with DAPI (Sigma; 1∶1000 in PBS 1X). Slides were mounted with Fluorsave (Calbiochem) and examined under a Zeiss AxioStar-Plus microscope. Images were collected with Zeiss 100× objectives.

### Two-dimensional Gel Electrophoresis (2-DE)

To perform isoelectric focusing, proteins (100 micrograms) were diluted in rehydration buffer (6 M urea, 2 M thiourea, 4% CHAPS, 0.02 M DTT, 0.5% IPG Buffer) to obtain a 125 µL final volume. Samples were loaded onto 7 cm linear pH 4–7 strips (Immoboline DryStrip, GE Healthcare). An Ettan IPGphor Isoelectric Focusing system (GE Healthcare) was used for the first dimension. The second dimension (10% NuPage Bis/Tris precast acrylamide gel) was performed on XCell SureLock*®* Mini-Cell system (Invitrogen). Coomassie Blue staining was used for image analysis and MS experiments.

### Image Acquisition and 2-D Gel Analysis

Coomassie Blue 2-DE gels were scanned and digitized using the GS-800 Calibrated Densitometer (Bio-Rad). Gel alignment, spot detection and quantification were performed using the PDQuest software (Sensitivity: 31.70, Size scale: 3, Min peak: 160, Bio-Rad). Data are the means of six gels from six different experiments.

### Mass Spectrometry

Maldi-TOF/TOF analysis of protein spot was performed. The target protein spot was excised from the stained gels, destained and denatured. The gel-piece was then incubated in digestion solution containing 20 µg/mL proteomics grade trypsin for 10–12 h at 37°C. The products of tryptic digestion were deposited on PAC (Prespotted AnchorChip) from Bruker Daltonics. The tryptic peptides were analysed using an autoflex III MALDI-TOF TOF equipped with a smartbeam laser and controlled by the FlexControl 3.0 and FlexAnalysis 3.0 software package. The mass spectrometer was operated with positive polarity in reflectron mode and spectra acquired in the range of m/z 500–4000. A timed ion gate was used for precursor ion selection and the fragments generated were further accelerated with 21 kV in the LIFT cell, and detected following passage through the reflectron. The used software was BioTools 3.2 (Bruker Daltonics). MS spectrum was performed in data-depended mode in which up to 4 precursor ions above an intensity threshold of 7 counts/s (cps) were selected for MS analysis from each survey “scan”. The tandem mass spectrometry data was converted into a PKL file using Masslynx v 4.0 software (Micromass, Waters, USA) and then was imported into a local Mascot 3.2 search engine. The search parameters in MatrixScience Mascot were as follows: the enzyme was trypsin; the taxonomy was selected as Homo sapiens; the mass tolerance was ±0.3 Da; the MS/MS tolerance was ±0.5 Da; the missed cleavage sites were allowed up to 1; the fixed modifications in NCBI nr were selected as carboxymethyl (cysteine); the variable modification was selected as oxidation (methylation) or none; the data format was selected as micromass PKL format; and the instrument was selected as MALDI-TOF/TOF. Individual ions scores >51 indicate identity or extensive homology (P<0.05). MS tolerance was 70 ppm.

### Surface Plasmon Resonance

Pure recombinant human calumenin was obtained from Prospec (Israel) and purified full length CFTR was a generous gift from Pr Robert Ford (University of Manchester, UK). CM5 Sensor chip, amine coupling kit (*N*-hydroxysuccinimide, (NHS), *N*-ethyl-*N*-(3-diethylaminopropyl) carbodiimide hydrochloride (EDC), ethanolamine (1 M, pH 8.5) and HBS-EP buffer (10 mM HEPES, 150 mM NaCl, 3 mM EDTA, and 0.005% surfactant P20 at pH 7.4) were obtained from GE Healthcare Bio-Sciences AB. The experiments were performed at the *PurIProb* core facility (Inserm, UMR1078, Brest). Real-time detection of the Calu - Wt-CFTR were performed using a Biacore system (Biacore 3000; GE Healthcare) and its Control Software version 3.2. All injections were performed at 25°C in HBS-EP 1× running buffer (GE Healthcare). Biacore 3000 was set at 25°C for all steps during the analytical process, and experimental data were collected at a medium rate. Biacore sensorgrams were analyzed using the BIAevaluation software. For each sample the obtained RU value was the value on the active flow cell (FC) minus the value of the reference FC, 20 seconds after the beginning of the dissociation phase. Binding of pure calumenin was performed according to Biacore’s recommendations on the CM5 sensor chips using an Amine Coupling Kit (GE Healthcare) to achieve about 2000 RU on FC2 and FC4, respectively. The surfaces were then blocked with 1 M ethanolamine hydrochloride (pH 8.5). The reference channels (FC1) were activated with equal volumes of NHS and EDC and immediately saturated with ethanolamine. Binding experiments were carried out at a flow rate of 5 µl/min using HBS-EP buffer pH 7.4 as running buffer, in triplicate, at 25°C. In between injections, the surface was regenerated by a 2 microliters pulse of 50 mM NaOH, which was found to be a suitable condition for removing the bound analyte on the immobilized antibody with a very low impairment of the ligands. The results presented here are from FC2-FC1. The reverse experiment in which CFTR was bound was also performed (not shown). Bovine Serum Albumin was used as a negative control. Affinity constants were calculated using the Biacore 3000 Control Software’s wizard (GE Healthcare).

### STRING Query of Protein Interaction Network

The calumenin - CFTR interaction which was identified experimentally was evaluated with the online Search Tool for the Retrieval of Interacting Genes/Protein (STRING 9.0, http://string-db.org) to predict both direct and indirect interactions [38]. The confident scoring of the interaction was based on known and predicted protein interactions collected from direct (physical) and indirect (functional) associations. The score was set at 0,4 which represents a medium confidence. The database quantitatively integrated interaction data from four sources - genomic context, high-throughput experiments, co-expression and previous knowledge from research publications. The results are presented with the input protein (calumenin) in the context of a graphical network of interaction partners. Associations in STRING are provided with a probabilistic confidence score which represents an estimate of how likely a given association describes a functional linkage between two proteins. The score is computed and indicates higher confidence when more than one type of information supports the association (direct physical interactions, genetic interaction, text mining, and experiments).

### Statistics

Results are expressed as means ± SEM for *n* cells. To compare data, Student *t* tests were performed. P<0.01 was considered statistically significant.
